# EGR2 affects mixed stroke repair through BNIP3L-mediated microglial mitophagy

**DOI:** 10.1016/j.ibneur.2026.04.002

**Published:** 2026-04-09

**Authors:** Xiujun Zhang, Bensi Zhang, Chun Shi, Natnicha Kampan, Waleephan Treebupachatsakul, Rungusa Pantan, Suteera Narakornsak, Manussabhorn Phatsara

**Affiliations:** a Department of Anatomy, Faculty of Medicine, Chiang Mai University, Inthawarorot Road, Chiang Mai, Thailand; b Department of Human Anatomy, College of Basic Medicine, Xiaguan Campus, Dali University, Wanhua Road, Dali City, Yunnan Province, China; c College of Dental Medicine, Western University of Health Sciences, Pomona, CA, USA

**Keywords:** Mixed stroke, Intracerebral hemorrhage, EGR2, BNIP3L, Mitophagy

## Abstract

Mixed stroke, also known as hemorrhagic infarction or infarction with hemorrhage, presents as a cerebral infarction combined with intracerebral hemorrhage (ICH) on computed tomography (CT) brain scans. ICH is a brain parenchymal hemorrhage caused by the loss of vascular integrity, which can lead to permanent disability or death. The early growth response 2 (EGR2) gene has been studied in a variety of brain diseases. However, effective treatments are still lacking. Methods: In this study, a cell model was constructed using oxyhemoglobin (OxyHb), and cell viability was detected using CCK-8. The mitochondrial membrane potential was measured using a mitochondrial membrane potential detection kit. Protein stress was used to assess the expression of EGR2, BCL2/adenovirus E1B 19 kDa protein-interacting protein 3-like‌ (BNIP3L or BNIP3L/NIX), and autophagy-related proteins. RT-qPCR detected the expression of EGR2 and BNIP3L mRNA. Microtubule-associated protein 1 A/1B-light chain 3 (LC3) expression was detected by immunofluorescence. Results: This study found that OxyHb reduced microglial viability in a concentration-dependent manner, and 20 μM OxyHb produced the most robust effect and was selected for subsequent experiments. In the cell model, the membrane potential of microglia decreased, and the fluorescence intensities of autophagy-related proteins (ATG7, LC3 II/LC3 I, and P62) and LC3 were inhibited. Over-expression-EGR2 (oe-EGR2) can increase the membrane potential of microglia and promote the fluorescence intensity of autophagy-related proteins (ATG7, LC3 II/LC3 I, and P62) and LC3. Mitochondrial division inhibitor-1 (Mdivi-1) and sh-BNIP3L could reverse the effect of oe-EGR2. Conclusion: EGR2 promotes microglial mitophagy by upregulating BNIP3L, thereby alleviating ICH.

## 1 Introduction

Mixed stroke, also known as hemorrhagic infarction or infarction with hemorrhage, presents as a cerebral infarction combined with intracerebral hemorrhage (ICH) on computed tomography (CT) brain scans (Craig et al., 2022, Ott et al., 1986).

Ischemic stroke is usually caused by an embolism of the middle cerebral artery, stenosis or occlusion of the extracranial internal carotid artery or vertebral artery, thrombosis, cerebral artery spasm, or cardiac arrest. Characteristically, ischemic strokes have a high incidence, disability rate, recurrence rate, and mortality rate, causing a severe economic and mental burden for the patients’ families. The pathological mechanism of ischemic stroke is complex. Cerebral vascular infarction leads to insufficient oxygen and sugar supply to the brain tissue, which then triggers a cascade reaction, including damage to the blood–brain barrier function, release of inflammatory cytokines, increased oxidative stress level, protein synthesis disorder, and abnormal mitochondrial metabolism, etc (Gou et al., 2021, Tuo et al., 2022, Galkin, 2019). Animal experiments have shown that using cell therapy containing bone marrow-derived mononuclear cells or macrophages can effectively alleviate the degree of brain injury in ischemic stroke model rats and improve the prognosis (Wang et al., 2013).

ICH is caused by a parenchymal hemorrhage due to a loss of vascular integrity, and is one of the most common subtypes of stroke (Li et al., 2022, Shi et al., 2020). After a cerebral hemorrhage injury, hematoma dissolution can cause a secondary brain injury (Ironside et al., 2019). It is reported that the 5-year mortality rate of patients is as high as 70%, and more than 80% of survivors face permanent disability (Ren et al., 2020). Neuronal apoptosis, white matter damage, and mitochondrial dysfunction are involved in hemorrhagic brain injury during the pathogenesis of ICH (Chen et al., 2020, Deng et al., 2021). Although scientists from various countries have conducted in-depth research on cerebral hemorrhage, they have not yet found an effective treatment. Large-scale randomized controlled clinical trials published both domestically and internationally have not shown that aggressive craniotomy for hematoma removal improves prognosis. Therefore, treatment of cerebral hemorrhage still requires endogenous mechanisms of hematoma removal (Mendelow et al., 2013).

In the diseased brain, microglial cells migrate to the damaged area and become activated. The branches of activated microglia become shorter and thicker, adopting an amoeboid shape. They engulf cell fragments or apoptotic cells and release various cytokines, exerting neuroprotective effects (Barath and Wu, 2025, Haruwaka et al., 2024). The inflammatory response mediated by microglia plays a crucial role in cerebral stroke and traumatic brain injury (Nan et al., 2022, Parusel et al., 2023, Navarro et al., 2018, Liu and McCullough, 2013). We hypothesize that microglial cell activation, their immune functions, and their interactions with other cells play a crucial role in the pathophysiological mechanisms underlying cerebral hemorrhage.

Autophagy is a process that engulfs damaged and dysfunctional organelles to maintain cellular homeostasis, including chaperone-mediated autophagy, microautophagy, and macroautophagy (Fleming et al., 2022, Li et al., 2021a). Mitophagy is autophagy that occurs in mitochondria and selectively identifies and eliminates dysfunctional or redundant mitochondria, thereby protecting the brain (Jiang et al., 2021). The decrease in oxygen metabolism in the hematoma after ICH leads to mitochondrial dysfunction. Autophagy is activated at 6 h after ICH and reaches its peak at 12 and 24 h (Zhang and Liu, 2020). The relationship between mitophagy and the maintenance of mitochondrial function is the pathological basis of ICH (Galluzzi et al., 2016). Therefore, regulating mitophagy to maintain mitochondrial integrity and homeostasis is the key to ICH treatment. However, the role of mitophagy in ICH remains unclear.

The early growth response 2 (EGR2) gene is located on chromosome 10q21.3 and is a transcription factor containing a zinc finger structure (Joseph et al., 1988). It has been reported that EGR2 is expressed in many tissues and different types of cells, and is closely related to inflammation, apoptosis, and tissue damage (Taefehshokr et al., 2017). At present, the research on EGR2 mainly focuses on tumors, myocardial dysfunction, and lupus-like autoimmune diseases (Bo et al., 2022, Dai et al., 2020, Ying et al., 2021). In addition, studies have shown that overexpression of EGR2 protects rat brains against ischemic stroke by downregulating the Ink signaling pathway (Niu et al., 2018). However, the role of EGR2 in cerebral hemorrhage remains unclear.

Since some mechanisms (Ren et al., 2020) and subsequent symptoms (Gou et al., 2021, Tuo et al., 2022, Galkin, 2019, Wang et al., 2013) following cerebral hemorrhage are similar to those of cerebral ischemia, the Oxygen–Glucose Deprivation (OGD) cell model was used in this study. The OGD cell model is an in vitro experimental model used to simulate ischemic brain injury. By removing oxygen and glucose from the cell culture environment, it reproduces pathological processes such as energy metabolism disorders, oxidative stress, and apoptosis in cells under ischemic conditions. Additionally, we selected commonly used parameters in cerebral hemorrhage, such as EGR2, BNIP3L, Autophagy-related protein 7 (ATG7), and P62, to study mixed cerebral hemorrhage. The main focus is to explore whether EGR2 affects mitochondrial autophagy in microglia and to further investigate the signaling pathways through which EGR2 influences it. The success of this study is expected to provide target genes for the clinical diagnosis and treatment of mixed stroke.

## 2 Materials and methods

### 2.1 Reagents

Mouse BV2 microglial cell line (Cat. #CL-0493A) was purchased from China Type Culture Center (Wuhan, Hubei, China). Modified Dulbecco’s modified Eagle’s high glucose medium (DMEM) medium (Cat# SH30243.01) and fetal bovine serum (FBS) (Cat# SH30070.03) were purchased from HyClone Inc. (USA). Oxyhemoglobin (OxyHb, #H8020) was purchased from Solarbio (Shanghai, China). Bovine Serum Albumin (BSA, Cat. #B14), Mitochondrial division inhibitor-1 (Mdivi-1, #47585610MG), and TRIzol™ reagent (Cat. #15596026CN) were purchased from Thermo Fisher Scientific China (Shanghai, China).Oe-EGR2 (#pGCMV), sh-EGR2 (#pGPU6), and sh-BNLP3 (#pGPU6) were designed, synthesized, and purchased from GenePharma (Shanghai, China). Microglial cells were transfected using Lipofectamine® 2000 reagent (#11668500) purchased from Seymour Fisher Technologies (Waltham, Massachusetts, USA). CCK-8 cell counting kit (#HY-K0301–120 mL) was purchased from MCE (Shanghai, China). EnzChek™ Reverse Transcriptase Assay Kit (#E22064), SYBR premix Ex Taq kit (#50–444–020), and the mitochondrial membrane potential detection kit (Image-iT™ TMRM Reagent #I34361) were purchased from Invitrogen (Shanghai, China). Primary antibodies were from Proteintech China (Wuhan, Hubei, China): LC3 (Cat. No. 18722–1-AP), ATG7 (10088–2-AP), p62 (18420–1-AP), EGR2 (13491–1-AP), BNIP3L (12986–1-AP), and GAPDH (10494–1-AP). For immunofluorescence, secondary antibodies CoraLite®488 goat anti-mouse IgG (H+L) (green; Cat. No. SA00013–1) and CoraLite®594 goat anti-mouse IgG (H+L) (red; Cat. No. SA00013–3) were purchased from Proteintech China (Wuhan, Hubei, China) and Abcam (Abcam China, Shanghai, China).

### 2.2 Cell culture and treatment

The mouse BV2 microglial cell line was cultured in Dulbecco’s modified Eagle’s high glucose medium supplemented with 10% FBS, 100 U/mL penicillin, and 100 μg/mL streptomycin. BV2 cells were seeded into a cell culture plate with serum-free medium 2 h before treatment with OxyHb. BV2 cells were then exposed to different concentrations of OxyHb (0, 5, 10, 15, and 20 μM) for 24 h to simulate ICH, and the control group was treated with an equal volume of phosphate-buffered saline (PBS). Finally, 20 μM OxyHb was used to construct the OGD cell model. Microglia were treated with Mdivi-1 before induction with OxyHb, as indicated by the grouping. Microglia were transfected with short hairpin RNA (sh-RNA), pcDNA3.1. Part of the cultivated cells were used as the NC (Normal cultured mouse BV2 microglial cells without undergoing cell modeling experiments) group and were employed as the control group for the experiment. In this study, the content presented in 3.1, 3.2 of the results section is described as the “NC” group.

### 2.3 Cell transfection

The oe-EGR2, sh-EGR2, and sh-BNLP3 used in this study, and the negative control sh-NC and oe-NC were designed and synthesized by GenePharma (Shanghai, China). Microglial cells were transfected with Lipofectamine® 2000 reagent, according to the manufacturer’s protocol. After 24 h of transfection, the cells were used for subsequent experiments. The negative control sh-NC and oe-NC groups contain empty vector plasmids. In this study, they were applied in 3.3, 3.4, 3.5 of the results section, and are referred to as the “NC” group.

### 2.4 CCK-8

The CCK-8 cell counting kit was used to assess BV2 cell viability. Cells treated with different concentrations of OxyHb (1 × 10^6^/well) were inoculated into 96-well plates, and then 20 μL CCK-8 solution was added to each well. After 2 h incubation, the absorbance was measured at 450 nm (A450) using a microplate reader (ELx800, USA). Cell viability = OD (treatment)/OD (control) x 100%.

### 2.5 Real-time PCR

After treatment with OxyHb for 24 h, RNA extracted using TRIzol reagent was reverse transcribed into cDNA using the EnzChek™ Reverse Transcriptase Assay Kit. The expression of EGR2 and BNIP3L was analyzed using the SYBR premix Ex Taq kit. The relative expression of the gene was analyzed by 2^-ΔΔCt^ using GAPDH as the internal reference gene.

### 2.6 Western blot

According to the reference, total protein from different cell groups was extracted for western blotting (Liao et al., 2020). The antibodies were purchased from Proteintech (ProteinTech China, Wuhan, Hubei, China) and Abcam (Abcam China, Shanghai, China). The primary antibody information is as follows: LC3 (1:25,000, 18420–1-AP), ATG7 (1:1500, 10088–2-AP), P62 (1:10,000, 18420–1-AP), EGR2 (1:2000, 13491–1-AP), BNIP3L (1:1500, 12986–1-AP), and GAPDH (1:20,000, 10494–1-AP). The blots were incubated with the primary antibodies at 4ºC overnight. Then, they were incubated with the corresponding secondary antibodies at room temperature (RT) for 1 h. GAPDH was used as the internal reference gene to calculate the relative expression of the protein.

### 2.7 Immunofluorescence

Cells from different groups were seeded on sterile glass coverslips. After reaching approximately 50% confluence, cells were washed three times with PBS and fixed with 4% paraformaldehyde for 15 min at RT. Cells were permeabilized with 0.1% Triton X-100 for 10 min and blocked with 5% BSA for 1 h at RT. Cells were then incubated with mouse anti-LC3 (1:200) at 4°C overnight. After three PBS washes, cells were incubated for 2 h at RT in the dark with fluorophore-conjugated secondary antibodies (green or red, 1:500; see Reagents). Nuclei were counterstained with DAPI for 2 min. Images were acquired using a fluorescence microscope (FV3000, Olympus, Tokyo, Japan).

### 2.8 Measurement of mitochondrial membrane potential

A mitochondrial membrane potential detection kit was used to detect microglial cell membrane potential. Briefly, cells were incubated with 5,5’,6,6’-Tetrachloro-1,1’, 3,3’-tetraethylbenzimidazolocarbo cyanine iodide (‌JC-1) at 37 °C for 30 min. Then, 500 mL PBS was added, and the cell fluorescence was observed using a fluorescence microscope (green fluorescence: Ex/Em = 510/527 nm; red fluorescence: Ex/Em = 585/590 nm). The ratio of red/green fluorescence intensity was inversely proportional to mitochondrial membrane potential.

### 2.9 Statistical analysis

All experiments were performed in at least three replicates. The experimental results are shown as mean ± standard deviation (SD). Statistical analysis was performed using GraphPad Prism 6 software. The comparison between the two groups was analyzed using a two-tailed *t*-test. Comparisons among the three groups were analyzed using ANOVA followed by Bonferroni/Dunn post hoc tests. A *p*-value < 0.05 was considered statistically significant.

## 3 Results

### 3.1 The expression of EGR2 and BNIP3L in a cell model

**Fig. 1:**
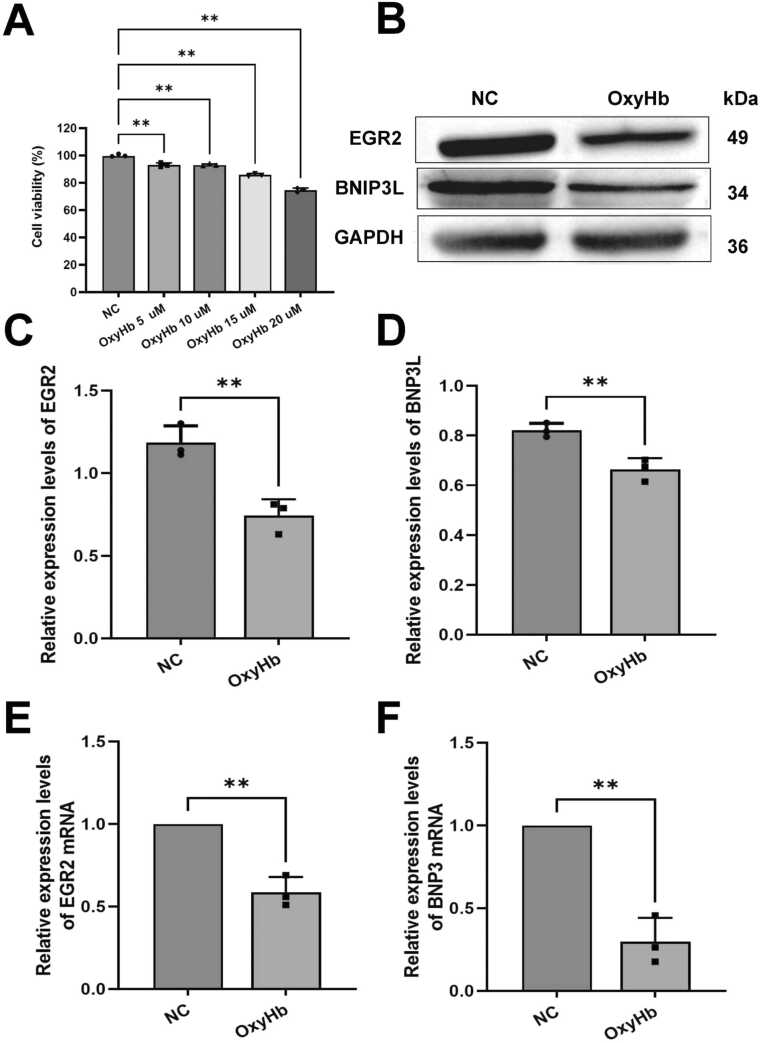
The expression of EGR2 and BNLP3L in the cell model. A: CCK-8 was used to detect cell viability. B: The expression of EGR2 and BNIP3L protein was detected by western blot. C and D: The expression of EGR2 and BNLP3L protein in Fig. 1B was quantified. E and F: The expression of EGR2 and BNIP3L mRNA was detected by RT-qPCR and quantified. The number of independent biological replicates for each experiment is 3. In summary, 20 μM OxyHb was selected to construct the cell model. Compared with the NC group, EGR2 and BNIP3L were highly expressed in the cell model; ** *p* < 0.01.

In this study, cell models were constructed using different concentrations of OxyHb (5, 10, 15, 20 μM). CCK-8 results showed that microglial viability was concentration-dependent with OxyHb, with cell viability reaching a critical value at 20 μM OxyHb. At this point, the cell vitality had decreased by more than 20%. To ensure that the cells maintain sufficient vitality during the experiment, 20 μM OxyHb was selected for ICH cell culture (Fig. 1A).

The expression levels of EGR2 and BNIP3L proteins were detected by western blot. The results showed that the expression of EGR2 and BNIP3L proteins was significantly decreased in the OxyHb group (Fig. 1B). The protein expression of EGR2 and BNIP3L (Fig. 1B) was significantly decreased, and the results were consistent (Figs. 1C and 1D). The results of RT-qPCR showed that mRNA expression of EGR2 and BNIP3L was significantly decreased in the OxyHb group (Figs. 1E and 1F). These results indicate that the expression of EGR2 and BNIP3L was reduced in the cell model.

### 3.2 Mitophagy of microglia was inhibited in cerebral hemorrhage

**Fig. 2:**
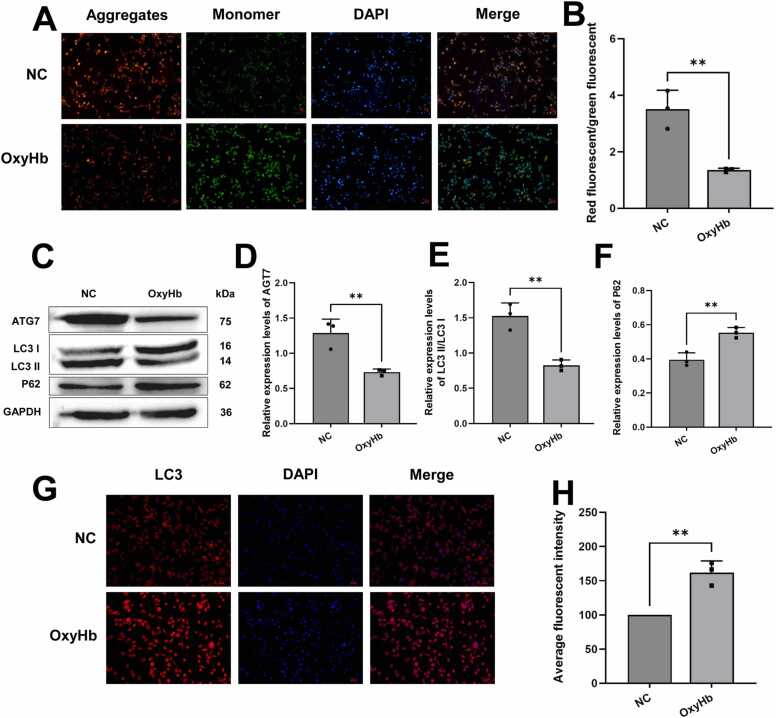
Mitophagy of microglia was inhibited in cerebral hemorrhage. A: Mitochondrial membrane potential of microglia decreased during ICH. Scale bar: 50 µm; B: A bar chart analysis of Figure A. C: Western blot detected a decrease in autophagy-associated proteins. D, E, and F: A bar chart analysis of Figure C. G: The expression of increased LC3 was detected by immunofluorescence. Scale bar: 20 µm. H: A bar chart analysis of Figure G. To present the true biological differences and data reliability rather than variations in laser power or technical errors, this experiment was repeated 3 times to ensure the results were reliable and reproducible. The conditions and outcomes of each experiment remained consistent, demonstrating the stability and reliability of the experimental results. In summary, compared with the NC group, microglial mitophagy was inhibited in ICH; **p < 0.01.

To explore whether microglial mitophagy is associated with ICH, we assessed mitochondrial membrane potential using immunofluorescence. It was observed that the JC-1 dye in the NC group primarily existed as aggregates. In contrast, the JC-1 dye in the OxyHb group mainly existed as monomers (Fig. 2A), indicating that the mitochondrial membrane potential of the cells decreased after OxyHb treatment (Fig. 2B).

Western blot analysis was performed to detect the expression of autophagy-associated proteins (ATG7, LC3 II/LC3 I, and P62). The structure showed that ATG7 and LC3 II/LC3 I were significantly reduced after OxyHb treatment, and p62 was increased, indicating a low level of autophagy at this time (Fig. 2C). ATG7 decreased, indicating that autophagy initiation was difficult (Fig. 2D). If the ratio of LC3 II to LC3 I decreases, LC3 II decreases and LC3 I increases. The decrease in LC3 II indicates that the number of autophagosomes is continuously decreasing. In contrast, the increase in LC3 I indicates that raw materials generated by autophagosomes are accumulating and that new autophagosomes are not being generated in substantial numbers (Fig. 2E). The increase in p62 indicates low autophagic flow (Fig. 2F). Immunofluorescence detection of LC3 expression also found that LC3 was significantly increased in the OxyHb group (Fig. 2G), indicating that the increase of LC3 I was very large, which exceeded the decrease of LC3 II (Fig. 2H). It also indicated that the generation of new autophagosomes decreased, and LC3 I, involved in the formation of new autophagosomes, was greatly accumulated, and old autophagosomes were dying. Mitochondria exhibit low levels of autophagy. In summary, microglial mitophagy was inhibited in ICH.

### 3.3 EGR2 can affect microglia mitophagy in cerebral hemorrhage

**Fig. 3:**
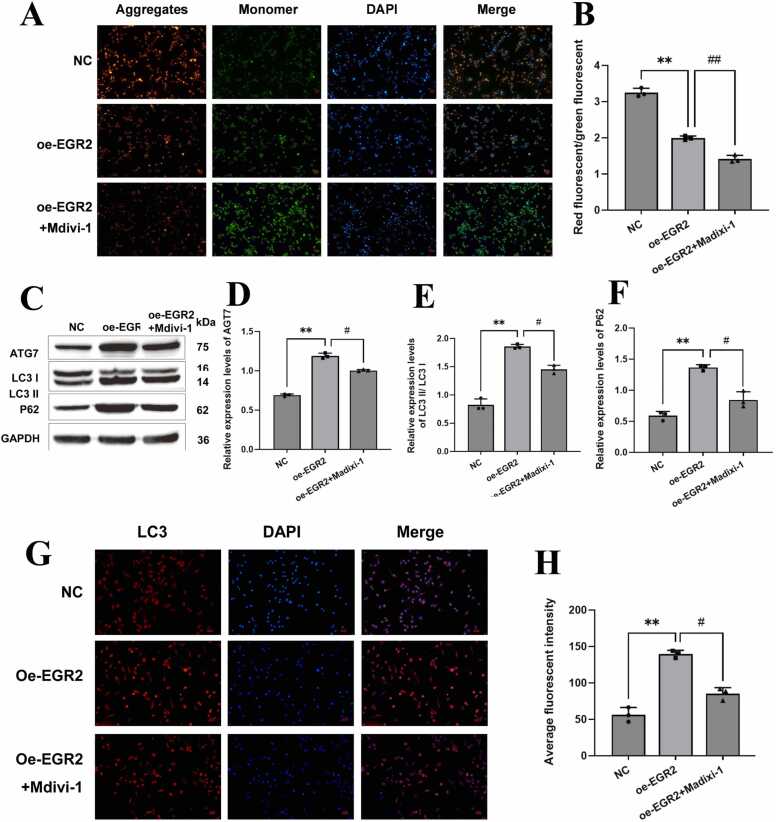
EGR2 can affect microglia mitophagy in cerebral hemorrhage. A and B: mitochondrial membrane potential detection kit to detect mitochondrial membrane potential. Scale bar: 50 µm. C, D, E, and F: Western blot was used to detect autophagy-related proteins. G and H: The expression of LC3 was detected by immunofluorescence. Scale bar: 20 µm. The number of independent biological replicates for each experiment is 3. In summary, compared with the NC group, EGR2 can promote microglial autophagy; ## *p* < 0.01, ** *p* < 0.01, # *p* < 0.05.

To investigate the role of EGR2 in microglia, we transfected ICH microglia models with oe-EGR2. Mitochondrial membrane potential detection showed that oe-EGR2 increased mitochondrial membrane potential, indicating increased mitochondrial energy production and a high-energy state. After the use of Mdivi-1, the effect of oe-EGR2 was significantly inhibited, and the mitochondrial membrane potential decreased, indicating that the energy produced by mitochondria at this time was reduced and the cell was in a low-energy state (Figs. 3A and 3B). Western blot results showed that oe-EGR2 could promote the expression of mitochondrial autophagy proteins ATG7, LC3 II/LC3 I, and P62. Increased ATG7 expression indicates mitochondrial autophagy has been activated. The ratio of LC3II/LC3I increased; that is, the expression of LC3II increased, and the expression of LC3I decreased, indicating that new autophagy was continuously generated, and the original autophagy was continuously digested. The increased expression of P62 indicates that the autophagy process was efficient and the autophagy flow was smooth. At this time, autophagy was elevated. After the use of Mdivi-1, the expression of the oe-EGR2 mitochondrial autophagy protein was significantly inhibited (Fig. 3C–F). LC3 fluorescence results showed that LC3 expression increased after oe-EGR2 treatment, that is, the increased amount of LC3 II was much greater than the decreased amount of LC3 I, indicating that autophagy activity was enhanced and regulated, autophagosomes were continuously generated and their functions improved, the mitochondrial quality of cells was regulated, and the number of mitochondria was reduced to the basic level. LC3 expression was inhibited after Mdivi-1 treatment (Figs. 3G and 3H). In summary, EGR2 can promote microglial autophagy.

### 3.4 EGR2 can regulate the expression of BNIP3L

**Fig. 4:**
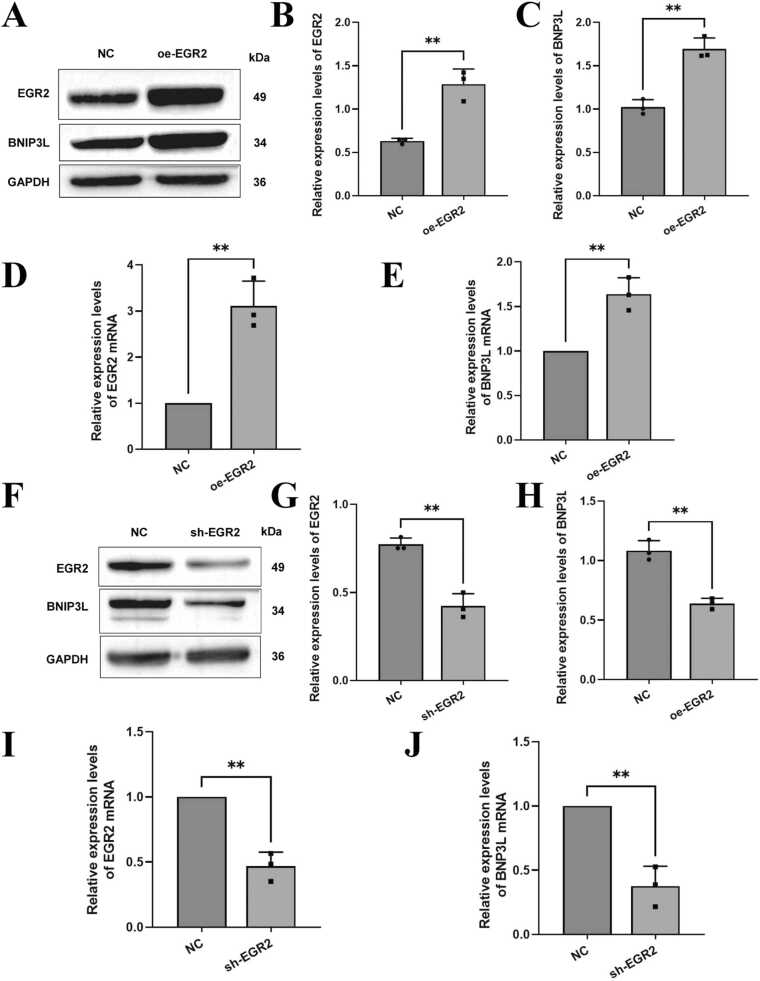
EGR2 can positively regulate BNIP3L expression. A, B, and C: After transfection with oe-EGR2, the protein expression of EGR2 and BNIP3L was detected by western blot. D and E: The mRNA expression of EGR2 and BNIP3L was detected by RT-qPCR. F, G, and H: After transfection with sh-EGR2, the protein expression of EGR2 and BNIP3L was detected by western blot. I and J: The mRNA expression of EGR2 and BNIP3L was detected by RT-qPCR. Compared with the NC group, ** *p* < 0.01. The number of independent biological replicates for each experiment is 3. In summary, compared with the NC group, EGR2 positively regulates BNIP3L expression.

To investigate whether EGR2 could regulate the expression of BNIP3L, EGR2 was transfected with oe-EGR2, and the protein and mRNA expression of EGR2 and BNIP3L were detected by western blot and RT-qPCR, respectively. The results showed that oe-EGR2 not only significantly increased protein expression of EGR2 and BNIP3L (Fig. 4A–C) but also significantly increased mRNA expression of EGR2 and BNIP3L (Figs. 4D and 4E). In addition, after transfection of EGR2 into cells with sh-EGR2, western blot results showed that sh-EGR2 could significantly inhibit the protein expression of EGR2 and BNIP3L (Fig. 4F–H). RT-qPCR results showed that sh-EGR2 significantly reduced mRNA expression of EGR2 and BNIP3L (Figs. 4I and 4J). In summary, EGR2 positively regulates BNIP3L expression.

### 3.5 EGR2 can affect microglia mitophagy through BNLP3L in cerebral hemorrhage

**Fig. 5:**
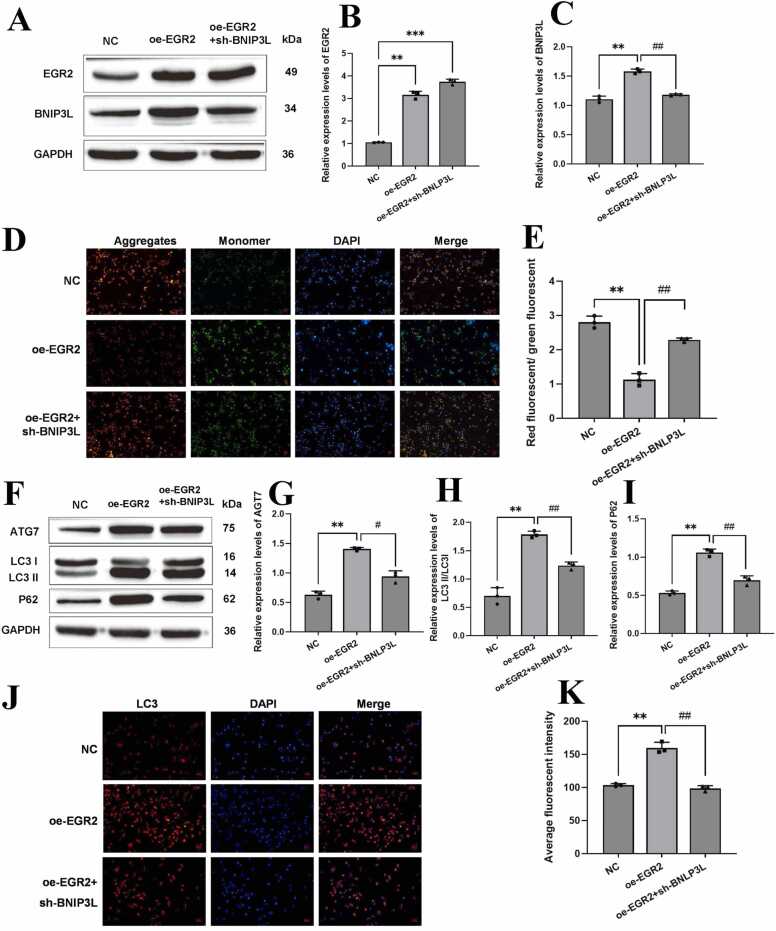
EGR2 can modulate microglial mitophagy via BNIP3L in cerebral hemorrhage. A, B, and C: The expression of EGR2 and BNIP3L protein was detected by western blot. D and E: mitochondrial membrane potential detection kit to detect mitochondrial membrane potential. Scale bar: 50 µm; F, G, H, and I: Western blot was used to detect autophagy-related proteins; J and K: The expression of LC3 was detected by immunofluorescence. Scale bar: 20 µm. Compared with the NC group, ** *p* < 0.01. Compared with the oe-EGR2 group, ## *p* < 0.01. The number of independent biological replicates for each experiment is 3. In summary, EGR2 can promote BNIP3L-mediated mitophagy in microglia.

Next, we investigated whether EGR2 could affect mitochondrial autophagy in microglia by regulating BNIP3L in an ICH model. In this step of the experiment, all NC groups were transfected with empty vector plasmids after the cells were successfully modeled. In Fig. 5, the western blot analysis of EGR2 and BNIP3L protein expression shows that, in the mixed cerebral infarction cell model, oe-EGR2 significantly increased the expression of both proteins. However, the oe-EGR2 + sh-BNIP3L treatment inhibited the expression of BNIP3L but had no significant effect on the expression of EGR2 (Fig. 5A–C). The mitochondrial membrane potential detection showed that oe-EGR2 could increase the mitochondrial membrane potential, while oe-EGR2 +sh-BNIP3L could inhibit the promoting effect of oe-EGR2 (Figs. 5D and 5E). Western blot analysis of autophagy-associated proteins showed that oe-EGR2 promoted the expression of ATG7, p62, and LC3 II/LC3 I, while oe-EGR2 + sh-BNIP3L reversed the effect of oe-EGR2 (Fig. 5F–I). At the same time, immunofluorescence assay of LC3 also obtained similar results (Figs. 5J and 5K). In summary, EGR2 can promote BNIP3L-mediated mitophagy in microglia.

## 4 Discussion

The proportion of patients with mixed stroke aphasia is relatively high, including aphasia and dysarthria, or both symptoms coexisting, which leads to communication difficulties and loss of social communication ability, seriously affecting the quality of life of the patients. Currently, in clinical practice, patients with mixed stroke have cerebral infarction and hemorrhage that mutually promote and transform each other, often presenting as advanced neurological functional impairments such as epilepsy, dementia, and aphasia (Finelli and DiMario, 2004). The treatment includes reducing intracranial pressure, adjusting blood pressure and blood viscosity, and taking hemostatic measures, which may have conflicting effects. It may lead to ischemia and at the same time increase hemorrhage, and vice versa, thus causing treatment contradictions (Pessin et al., 1993).

ICH is a disease with high mortality and disability (Nobleza, 2021). In the pathogenesis of ICH, the destruction of glial and neuronal cells leads to neurotransmitter release, membrane depolarization, oligoemia, and mitochondrial dysfunction (Lusardi et al., 2004, Qureshi et al., 2003). Microglia are activated in ICH and cause secondary damage such as edema, inflammation, and destruction of the blood–brain barrier in the area around the ICH (Hua et al., 2006, Yang et al., 2006), thereby changing motor function, cognitive, and emotional behavior (Zhu et al., 2018). Therefore, we believe microglia are key to the treatment of cerebral hemorrhage. In this study, we found that OxyHb had a dose-dependent effect on microglia survival, with 20 μM OxyHb having the best modeling effect on microglia. Given the similar mechanisms (Ren et al., 2020) and subsequent symptoms (Gou et al., 2021, Tuo et al., 2022, Galkin, 2019, Wang et al., 2013) following cerebral hemorrhage, this study used the OGD cell model to simulate an in vitro model of ischemic brain injury. Additionally, EGR2, BNIP3L, ATG7, and P62 were selected as commonly used parameters in cerebral hemorrhage to study mixed cerebral hemorrhage and to explore in depth whether EGR2 affects mitochondrial autophagy in microglia and the related signaling pathways influenced by EGR2. Under hypoxic conditions, EGR2 and BNIP3L expression decreased, mitochondrial membrane potential decreased, and autophagy protein expression decreased. At this time, autophagy was low, indicating that microglial autophagy was inhibited during ICH.

Mitochondria are involved in various cellular functions (Liu et al., 2018). The process of eliminating excess or dysfunctional mitochondria via autophagy to regulate mitochondrial numbers and maintain cellular homeostasis is called mitophagy (Bock and Tait, 2020, Youn et al., 2022). Appropriate mitophagy is of great significance for cell development and human diseases (Li et al., 2021b). Studies have shown that mitochondrial function is also important for neuronal health (Wang et al., 2018). The activity of axon and dendritic neuron fibers is highly dependent on mitochondria (Huang and Jiang, 2019). Mitochondrial damage can increase intracellular reactive oxygen species production, significantly decrease ATP levels, and cause Ca^2+^ overload, all of which are important factors in exacerbating ICH-induced secondary brain injury (Bian et al., 2025, Guan et al., 2018). Therefore, mitophagy is essential for neuronal survival after ICH. In this study, we found that the mitochondrial membrane potential of microglia was significantly decreased after OxyHb induction, and that autophagy-related proteins and LC3 fluorescence were decreased.

EGR2 is a transcription factor that has been found to induce cancer cell apoptosis by altering mitochondrial membrane permeability, releasing cytochrome C, and activating apoptosis-related proteins (Unoki and Nakamura, 2003). EGR2 may play an important role in the host defense of the immune response in the central nervous system, in which microglia are important immune cells (Yan et al., 2013). In addition, EGR2 is a key factor in age-dependent susceptibility to sevoflurane-induced cognitive impairment (Chen et al., 2022). In this study, it was found that when oe-EGR2 was transfected into the ICH model, mitochondrial membrane potential increased, indicating increased mitochondrial energy production and increased expression of an autophagy protein. These findings suggest that EGR2 can promote microglial autophagy.

As a mitochondrial outer membrane protein, BNIP3L has been found to induce mitophagy in natural killer cells, neurons, retinal ganglion cells, renal cells, and several types of tumor cells (O’Sullivan et al., 2015, Xu et al., 2019, Yuan et al., 2017). The presence of BNIP3L leads to the loss of mitochondrial transmembrane potential, which is considered to be a marker of dysfunction that triggers mitophagy (Chen et al., 2010, Diwan et al., 2009). In this study, EGR2 positively regulated BNIP3L expression. EGR2 promoted autophagy of nerve cells and alleviated cerebral hemorrhage by enhancing the expression of BNIP3L. At the same time, oe-EGR2 can promote microglial autophagy, and sh-BNIP3L can reverse the effect of oe-EGR2.

## 5 Conclusion

In this study, we found that EGR2 promoted microglial autophagy by increasing BNIP3L expression. Additionally, this study, for the first time, linked EGR2 and BNIP3L to ICH as a strategy to enhance the neuroprotective effect in the mixed stroke model.

## Funding

This study was supported by the 10.13039/501100010731Faculty of Medicine, Chiang Mai University (ANA-2567–0637) and universities granted by the Department of Science and Technology of Yunnan Province (NO.202101AN070028) and the Li Yunqing expert workstation of Yunnan Province (No.202005AF150014).

## CRediT authorship contribution statement

**Waleephan Treebupachatsakul:** Writing – review & editing. **Natnicha Kampan:** Writing – review & editing. **Chun Shi:** Writing – review & editing. **Bensi Zhang:** Writing – review & editing, Investigation, Data curation. **Manussabhorn Phatsara:** Writing – review & editing, Supervision, Funding acquisition, Conceptualization. **Suteera Narakornsak:** Writing – review & editing. **Rungusa Pantan:** Writing – review & editing. **Xiujun Zhang:** Writing – review & editing, Writing – original draft, Visualization, Validation, Methodology, Investigation, Formal analysis, Conceptualization.

## Conflicts of Interest

The authors declare that they have no known competing financial interests or personal relationships that could have appeared to influence the work reported in this paper.
